# Editorial: Trends in Muscle and Tendon Molecular and Cell Biology

**DOI:** 10.3389/fphys.2021.832613

**Published:** 2022-02-03

**Authors:** Rita de Cássia Marqueti, Michael Kjaer, Anselmo Sigari Moriscot

**Affiliations:** ^1^Graduate Program in Rehabilitation Sciences and Graduate Program of Sciences and Technology of Health, Faculty of Ceilândia, University of Brasília, Brasília, Brazil; ^2^Department of Orthopedic Surgery, Institute of Sports Medicine, Copenhagen University Hospital - Bispebjerg Frederiksberg, and Center for Healthy Aging, Faculty of Health and Medical Sciences, University of Copenhagen, Copenhagen, Denmark; ^3^Department of Anatomy, Institute of Biomedical Sciences, University of São Paulo, São Paulo, Brazil

**Keywords:** skeletal muscle, tendon, myotendinous junction, extracellular matrix, mechanosignaling, basic science

## Introduction

The musculoskeletal system performs a vital role over life, participating in structural support, delicate to complex movements involved in daily life activities, elite sporting events, and body protection/stabilization (Greising et al., 2020). The musculoskeletal system includes muscles and tendons, as well as ligaments and bones (Yamamoto and Abe, 2020). Muscles and tendons are fundamental for locomotion and posture maintenance, however, although they have been largely explored at the structural, functional, and cellular/molecular biology levels, their adaptive mechanisms are still largely unknown. The connection site between muscle and tendon is named the myotendinous junction (MTJ), which is a fundamental transitional structure for transferring muscle force from the contractile apparatus to the tendon, and ultimately to the final destination: bone, cartilage, skin, and eye (Charvet et al., 2012). Further, the MTJ is the most prominent place for traumatic muscle strain injury. This will put it into perspective also of sports injury. Considering this critical issue, the overall aim of this Research Topic was to investigate how muscle, tendon, and the myotendinous junction adapt under different stimuli conditions, such as mechanical unloading (atrophy) and loading (exercise), sepsis, inflammation, lesion, and aging, under the perspective of basic science. Another goal was to characterize the role of the extracellular matrix (ECM), matrix cell interactions with molecular pathways, and morphological tissue properties under these conditions. In this Research Topic we will present a collection of studies on skeletal muscle (seven articles) and tendon (two articles), and one study specifically addressing the muscle-tendon junction. Researchers in the area can benefit from the advancement in the science and increased chances of integrative insights into muscle and tendon.

## Muscle

Skeletal muscle tissue contains immense, highly specialized cells with an exceedingly developed cytoskeleton, where myosin interacts with actin, generating mechanical energy. Furthermore, skeletal muscle is particularly abundant, comprising 40–60% of total body mass in humans, and is involved in body mobility and stands as an essential energy reservoir (Frontera and Ochala, 2015). It is now clear that decreased skeletal muscle mass is an independent predictor of mortality (Du et al., 2014). Therefore, deep understanding of the biology of muscle atrophy is essential for the future development of more efficient treatments, especially in older adults, which is one of the focuses of this Research Topic: He et al. revealed the circulating microRNAs involved in older adults with sarcopenia. In addition a comprehensive time course of the transcriptome in the clinically relevant rotator cuff tear model, including intense atrophy was reported by Vasquez-Bolanos et al. Diaphragm muscle is especially prone to develop atrophy and quick functional loss under stressful conditions such as sepsis and muscular dystrophies. In this collection, it was emphasized that diaphragm under sepsis undergoes functional loss through mitochondrial dysfunction (Oliveira et al.). Furthermore, it was showed that oxidative stress is a key player in diaphragm degeneration, which can be mitigated by anti-oxidant treatment (Silva et al.). Physical exercise is certainly one of the most powerful strategies to fight skeletal muscle atrophy. Physical activity could create an intergenerational drive, attenuating the harmful effects of a high fat diet (HFD) in skeletal muscle from offspring (Salomão et al.). As demonstrated by Rovina and coauthors, even a single bout of physical activity can boost the expression of Nr1d1, a nuclear factor known to decrease atrophy gene program and increase mitochondrial biogenesis (Rovina et al.).

## Tendon

Of all consultations performed by primary care physicians, 20% are related to musculoskeletal diseases; 30% of these are associated with tendon injuries, called tendinopathies, which represent a highly prevalent problem in musculoskeletal medicine (Jordan et al., 2014). According to Burton, an important range of physical, genetic, and psychological individual factors has been recognized in patients affected by tendinopathy, resulting in a tremendous heterogeneous population and a challenge for rehabilitation (Burton). For this reason, understanding the basic science of tendons such as tendon homeostasis, maintenance, remodeling, and repair is an essential strategy for improving rehabilitation. Accordingly, we know that mechanical load is vital for tendon development and homeostasis to preserve all properties of the extracellular matrix (ECM). Tendons are mechanosensitive soft tissues able to transmit tensile forces between muscle and bone, acting in a catapult-like manner, also called a mechanical spring mechanism, by storing and releasing energy and then increasing the efficiency of the movement system (Nakajima et al., 2021). It is well-known that tendon fibroblasts adapt to the mechanical forces, transmitting forces across the extracellular matrix (ECM) without losing mechanical dynamism (Sawadkar et al., 2020). The specific ECM enables joint function and mediates mechanical signals to tendon cells, driving biological responses to exercise or injury. Interactions between the cells and the matrix (cell-matrix interactions) permit tenocytes (predominant cell type in the tendon) to sense and reply to mechanical signals with a catabolic or anabolic response (Kjaer, 2004; Chatterjee et al., 2021). Mechanotransduction is an essential mechanism by which mechanical stress acts upon a cell, initiating intracellular signaling, promoting cell growth and survival. As a tissue that efficiently transmits the mechanical forces, it is essential to comprehend the mechanisms of mechanotransduction. New insights have been gained in which several ion channels and receptors are identified as mechanosensors. The mechanosensitive ion channel PIEZO1 is responsible for different mechanotransduction processes in the lymphatic, cardiovascular, renal, and skeletal systems. Passini et al. (2021) analyzed the effect of reduced and elevated PIEZO1 mechanosignaling in tendons of rodents. *Piezo1* knockout mice (*Piezo1*cKO) showed reduced tail tendon stiffness, while *in vitro* pharmacological PIEZO1 stimulation and *in vivo* PIEZO1 overactivity improved tendon stiffness and strength (Passini et al., 2021). Mechanical properties allow tendons to respond and adapt to the load transmitted by the muscles. Collagen fibrils are tendon force-transmitting units distributed within the ECM and oriented parallel to the bone-muscle axis (Eekhoff et al., 2021). Tension across the muscle and tendon is essential for maintaining the integrity of both tissues. When one of them undergoes a change, both are affected. In this collection, it was reported that transcriptional changes in supraspinatus muscle after a rotator cuff tear model in rabbits. Consequently, the dysfunction led to muscle atrophy and fatty infiltration (Vasquez-Bolanos et al.). However, the transmitting force generated by the muscle to the tendon depends on the connection between these tissues, the MTJ.

## Myotendinous Junction

The MTJ is a specific anatomical region connecting skeletal muscle to tendon. The MTJ constitutes an integrated mechanical unit, the major site of force transmission. The muscle cell membrane is folded into finger-like extensions and invaginations to increase the area and allow the membrane to resist muscle contraction forces (Charvet et al., 2012; Subramanian and Schilling, 2015). The significant molecules of the tendon side are collagen I and tenascin-care, while the laminins and collagen IV are the major constituents of the muscle side (Subramanian and Schilling, 2015). Jakobsen and Krogsgaard, presented in this collection a review exploring the importance of MTJ in the sports field since the majority of strains are most dominantly located at the MTJ. The MTJ adapts to load by increasing the remodeling and junctional interface, while the opposite effect, shortening the folding in a smaller interface, occurs during inactivity. The authors also concluded that the limited knowledge about human MTJ is related to the lack of a standard method to obtain biopsies from this region. The MTJ is an essential source of information about structural proteins. However, investigations focusing on other molecules such as macrophages, satellite cells, fibroblasts, and adipocytes are necessary, since these molecules are essential for remodeling following exercise, injury, and inactivity.

## Conclusion

This Research Topic mainly highlights the critical role of mechanical load in maintaining the integrity of muscle, tendon, and the connection site between these tissues (MTJ). Most of the studies published on this Research Topic referred to muscle tissue. However, forthcoming studies need to address additional aspects, such as the mechanisms of molecular and cellular pathways in response to tendon and skeletal muscle in a wide-ranging situation such as injury; chronic and acute responses to loading, unloading, and aging; how the muscle-tendon unit adapts to inactivity and immobilization in combination with injury and aging, and with particular attention to the interactions of these mechanisms in MTJ.

## Author Contributions

RM, MK, and AM: study conception and design and draft manuscript preparation. All authors listed have made a substantial, direct, and intellectual contribution to the work and approved it for publication.

## Conflict of Interest

The authors declare that the research was conducted in the absence of any commercial or financial relationships that could be construed as a potential conflict of interest.

## Publisher's Note

All claims expressed in this article are solely those of the authors and do not necessarily represent those of their affiliated organizations, or those of the publisher, the editors and the reviewers. Any product that may be evaluated in this article, or claim that may be made by its manufacturer, is not guaranteed or endorsed by the publisher.
